# East Meets West: Developing Support Systems to Meet the Diverse Needs of Older Adults in the United States and in China

**DOI:** 10.1093/geroni/igaa057.1946

**Published:** 2020-12-16

**Authors:** Bei Wu, Jiehua Lu

## Abstract

With the rapid growth of the aging population around the world, developing support systems for older adults has become increasingly important. It is crucial for researchers， educators, policy makers to share their experience and knowledge to initiate innovative and supportive programs and services that will meet the challenges of the aging population. The East meets West Forum is a platform that researchers from the Gerontological Society of America and the Chinese Association for Gerontology and Geriatrics established in 2017. Previously, the East meets West Forum focused on the issues of the long-term care (LTC) workforce, LTC services, and programs for older adults in the U.S and in China. In this session, we include four presentations (two from the U.S. and two from China) that focus on a broader area of support systems, beyond LTC, that would meet the diverse needs of older adults from housing, wellness visits, family caregiving system, to end of life care. More specially, it includes: 1) expand housing services for low-income older adults; 2) strengthen family support systems and promote intergenerational support; 3) develop a comprehensive program for early detection and treatment of dementia at primary care settings; and 4) examine diversity in the family care patterns for the oldest old. This session provides opportunities for aging researchers/educators from two countries to share their knowledge and experience on developing supportive systems for older adults and their families. It also provides policy discussions on improving health and family caregiver support services in these two countries.

